# Automated multimodel segmentation and tracking for AR-guided open liver surgery using scene-aware self-prompting

**DOI:** 10.1007/s11548-025-03381-6

**Published:** 2025-05-14

**Authors:** Serouj Khajarian, Michael Schwimmbeck, Konstantin Holzapfel, Johannes Schmidt, Christopher Auer, Stefanie Remmele, Oliver Amft

**Affiliations:** 1 Research Group Medical Technologies, University of Applied Sciences Landshut, 84036 Landshut, Germany; 2 Intelligent Embedded Systems Lab., University of Freiburg, 79085 Freiburg, Germany; 3 LAKUMED Kliniken, 84036 Landshut, Achdorf, Germany; 4 Hahn-Schickard, 79110 Freiburg, Germany

**Keywords:** AR-guided surgery, Open liver surgery, Segmentation, Tracking

## Abstract

**Purpose:**

We introduce a multimodel, real-time semantic segmentation and tracking approach for Augmented Reality (AR)-guided open liver surgery. Our approach leverages foundation models and scene-aware re-prompting strategies to balance segmentation accuracy and inference time as required for real-time AR-assisted surgery applications.

**Methods:**

Our approach integrates a domain-specific RGBD model (ESANet), a foundation model for semantic segmentation (SAM), and a semi-supervised video object segmentation model (DeAOT). Models were combined in an auto-promptable pipeline with a scene-aware re-prompting algorithm that adapts to surgical scene changes. We evaluated our approach on intraoperative RGBD videos from 10 open liver surgeries using a head-mounted AR device. Segmentation accuracy (IoU), temporal resolution (FPS), and the impact of re-prompting strategies were analyzed. Comparisons to individual models were performed.

**Results:**

Our multimodel approach achieved a median IoU of 71% at 13.2 FPS without re-prompting. Performance of our multimodel approach surpasses that of individual models, yielding better segmentation accuracy than ESANet and better temporal resolution compared to SAM. Our scene-aware re-prompting method reaches the DeAOT performance, with an IoU of 74.7% at 11.5 FPS, even when the DeAOT model uses an ideal reference frame.

**Conclusion:**

Our scene-aware re-prompting strategy provides a trade-off between segmentation accuracy and temporal resolution, thus addressing the requirements of real-time AR-guided open liver surgery. The integration of complementary models resulted in robust and accurate segmentation in a complex, real-world surgical settings.

## Introduction

Although laparoscopy advances, open liver surgery remains an essential procedure due to tumor location, procedural complexity, patient history, etc. [1]. In open liver surgery, intraoperative ultrasound is the primary method used by surgeons to visualize critical structures of the liver. However, intraoperative ultrasound lacks 3D visualization and thus increases surgery duration and the risk of incomplete tumor removal [2].

Augmented Reality (AR) techniques could reduce intervention time and complications, but are mainly applied in orthopedic surgery so far [3]. AR guidance in liver surgery poses unique challenges, including real-time tracking. The non-rigid liver requires continuous surface tracking to account for deformations during registration [2] and thus limits the applicability of traditional marker-based tracking methods [4]. Simultaneous Localization and Mapping (SLAM) has been explored for liver tracking in laparoscopic surgery, where constrained camera viewpoints and pose estimation are used to reconstruct the liver surface [5]. In open liver surgery, a larger liver portion is visible than in laparoscopic surgery, thus reducing the need for reconstructions, e.g., SLAM. A continuous full-surface segmentation is necessary to handle large-scale deformations. Current segmentation and tracking techniques for open liver surgery often require user interaction and multiple devices, which complicates clinical workflows [2, 6].

Recently, deep learning approaches have gained attention for the segmentation and tracking of anatomical structures. In laparoscopic surgeries, reference datasets have been used to develop segmentation techniques for surgical videos [7]. Beyond surgical applications, recent studies have highlighted the potential of deep learning models for real-time RGBD segmentation. The Efficient Scene Analysis Network (ESANet) model was designed for real-time applications that require semantic segmentation and has demonstrated state-of-the-art segmentation performance on various RGBD benchmark datasets at an inference speed of up to 35 frames per second (FPS) [8]. However, supervised segmentation models, like ESANet, require a considerable amount of annotated data. Specifically, for liver segmentation and tracking in open liver surgery, there are no annotated data sets, which limits the applicability of supervised models.

Semi-supervised and zero-shot video object segmentation models have exhibited promising potential in mitigating the scarcity of annotated data. Recently, decoupling features in hierarchical propagation and associating objects with Transformers (DeAOT) has achieved state-of-the-art performance on benchmark datasets [9]. DeAOT supports real-time segmentation at rates of 12-64 FPS, depending on the dataset and encoder. However, the aforementioned models rely on annotated reference frames for initialization, which is impractical in real surgical scenarios.

Image segmentation foundation models, e.g., the Segment Anything Model (SAM) [10], which is trained on an extensive dataset (11 million images) have demonstrated zero-shot segmentation capabilities on unseen data. SAM has often outperformed fully supervised methods on a variety of segmentation datasets. Preliminary work integrated DeAOT and SAM for interactive and automatic segmentation in pre-recorded videos [11]. Although semi-supervised and zero-shot methods mitigate data scarcity issues, they remain dependent on user input via prompts or reference annotations, limiting their utility in applications requiring continuous segmentation like AR-guided liver surgery.

In a previous study, we introduced a pipeline for real-time organ tracking and registration in AR-guided liver surgery, validated in a phantom study [12]. The pipeline processes RGBD data from a head-mounted AR device, eliminating the need for markers and additional sensors. The RGBD data were segmented to create a 3D point cloud of the liver surface, which was subsequently used for registration. Yet, the feasibility of fully automated real-world image-based liver segmentation and tracking with limited training data has not been evaluated. The contributions of this work are:

1. We propose a novel multimodel approach for continuous liver segmentation and tracking in AR-guided open liver surgery that combines ESANet, SAM, and DeAOT to eliminate manual interaction by automated re-prompting.
2. We introduce automated re-prompting methods that use RGBD images, segmentation maps, and camera poses to detect changes in the surgical scene. Re-prompting can reduce segmentation errors due to occlusions, deformations, and camera movements. Additionally, we performed a trade-off analysis between segmentation accuracy and temporal resolution.
3. We evaluated our approach in a patient study. In particular, we collected data during open liver surgeries using Microsoft HoloLens2. In addition, we performed a comparative analysis of state-of-the-art segmentation methods based on our surgery dataset.

## Methods

**Fig. 1:**
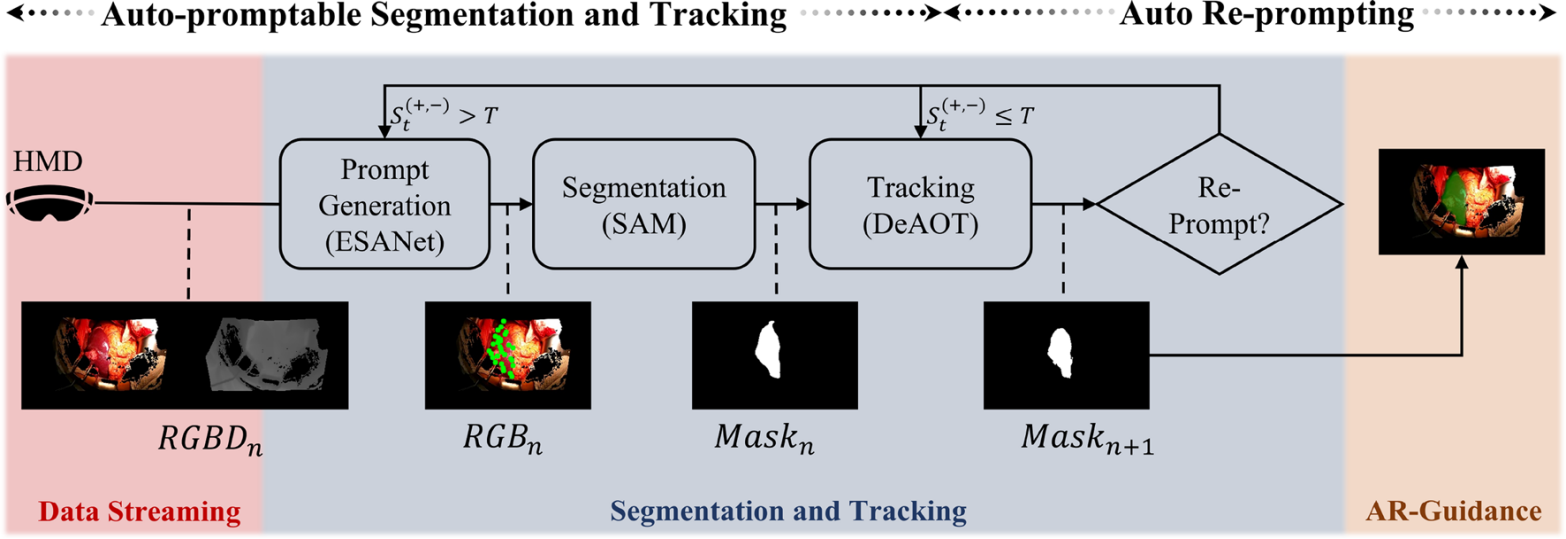
Our multimodel approach for AR-guided liver surgery combines: (1) An auto-promptable segmentation pipeline using ESANet for prompt generation, SAM for semantic segmentation, and DeAOT for subsequent frame tracking; (2) Automated re-prompting, including interval-based and scene-aware strategies. Our scene-aware re-prompting triggers, when upper or lower CUSUM values $S_t^{(+,-)}$ exceed a dynamic threshold $T$

Our multimodel approach integrates (1) an auto-promptable segmentation and tracking pipeline and (2) an adaptive re-prompting algorithm to balance processing complexity and performance under varying surgical conditions. Figure 1 provides an overview of our approach.

### Auto-promptable segmentation and tracking

Our pipeline integrates ESANet, SAM, and DeAOT models for liver segmentation and tracking to take advantage of both RGB and depth data. Our segmentation and tracking pipeline has three stages: prompt generation, segmentation, and tracking.

**Prompt Generation.** The ESANet model [8] processes the RGBD frame from the HoloLens2 sensors. ESANet was selected for its efficiency in handling both RGB and depth data simultaneously. Initially, pre-trained on the SceneNet dataset [13], ESANet was fine-tuned with our clinical RGBD data. Fine-tuning included updating output layers with pre-trained features, followed by full network adaptation. Data augmentation involved random rotations, flips, and contrast changes. ESANet generates an initial segmentation map to assign class labels and probability scores to each pixel. Pixels with segmentation probabilities above 90% were chosen as candidate prompt points to maximize prompt points within liver boundaries. To determine the placement of prompt points within the segmented area, the spacing between the prompt points $s$ was derived as:

1 $$\begin{aligned} s = \frac{\sqrt{N}}{f}, \end{aligned}$$

where $$N $$ is the number of candidate points and $$f $$ is a predefined spacing factor ($$f $$= 0.1).

**Segmentation.** The generated prompt points and the RGB image are passed to the Segment Anything Model (SAM) [10] to obtain a detailed segmentation of the liver region. We take advantage of the zero-shot segmentation capability of SAM, which is especially advantageous for limited labeled data. To maximize accuracy, we use the largest SAM variant, ViT-H (632 million parameters). The input images were resized and padded to the SAM designated resolution of 1024 $\times$ 1024 pixels. SAM then produces a refined binary segmentation mask that outlines the liver region, which is rescaled to match the original image resolution.

**Tracking.** The binary liver segmentation mask serves as a reference to track subsequent video frames with DeAOT [9]. The long- and short-term attention mechanisms of DeAOT handle scene variability, making it suitable for dynamic surgical environments. We used the best-performing variant (SwinB-DeAOTL), which was trained on large datasets, including ImageNet [14], DAVIS [15], and YouTube-VOS [16]. DeAOT propagates the segmentation across subsequent RGB frames.

### Auto re-prompting

To mitigate segmentation and tracking errors arising from factors including liver deformations, camera movements, occlusions, or significant scene changes [9], two re-prompting algorithms were implemented: interval-based re-prompting and scene-aware re-prompting.

**Interval-Based Re-Prompting.** The interval-based re-prompting method re-initializes the segmentation process at fixed frame interval *n*, independent of scene changes.

**Scene-Aware Re-Prompting.** Scene-aware re-prompting considers scene-related parameters (e.g., the consistency of the segmentation map) to identify deviations that could influence the tracking performance. Upon detecting a parameter deviation, SAM is re-prompted using the domain-specific ESANet model. Thus, the pipeline is re-initialized to sustain segmentation performance while minimizing inference time by activating SAM only when necessary.

To detect deviations, we use the cumulative sum (CUSUM) method [17]. The deviation $$ dX_t$$ was determined as the difference between observed $$X_t$$ and target values $$ \mu _t$$, where the target value $$ \mu _t $$ was obtained as the moving average between the five most recent frames. Subsequently, the upper and lower CUSUM values were updated as:

2 $$\begin{aligned} S_t^+&= \max \left( 0, S_{t-1}^+ + dX_t - k \sigma _t \right) \end{aligned}$$

3 $$\begin{aligned} S_t^-&= \max \left( 0, S_{t-1}^- - dX_t - k \sigma _t \right) , \end{aligned}$$

where $$S_t^+ $$ and $$S_t^- $$ are upper and lower cumulative sums that capture the extent of positive and negative parameter deviations, respectively. Parameter $$k $$ adjusts the sensitivity to deviations and is typically set to $$k=0.5$$ to balance the sensitivity of deviation detection with the frequency of prompting [18]. With $$ \sigma _t $$, we denote the standard deviation of the last five frames. A change is detected when either the upper or lower CUSUM value $$S_t^{+,-}$$ exceeds a threshold $$T $$, which was defined as:

4 $$\begin{aligned} T = \alpha \cdot \sigma _t. \end{aligned}$$

In Eq. (4), $$\alpha $$ controls the sensitivity of threshold $$T $$ (re-prompting sensitivity).

We evaluated a variety of scene-related parameters and values of $\alpha$ to determine performance effects, including segmentation accuracy (IoU) and temporal resolution (FPS).

### Evaluation methodology

#### Clinical data acquisition

We collected 20 recordings from 10 patients who underwent open liver resection surgery. To ensure that the AR video data are diverse, we recorded each patient from both sides of the surgical field. The videos were captured from the surgeon’s perspective using a HoloLens2 headset (approx. 2.5 min duration per recording). Video capture began after surgical intervention had started and right before the resection phase. To ensure that the captured data closely resembled the actual surgical conditions, surgeons were instructed to perform typical resection preparation during video recording. Liver visibility in the recordings varied due to occlusions, camera movements, and changes in the field of view (FOV). HoloLens2 data (including RGB and depth images, and camera poses) were streamed via the hl2ss application [19] over a wireless network.

**Fig. 2:**
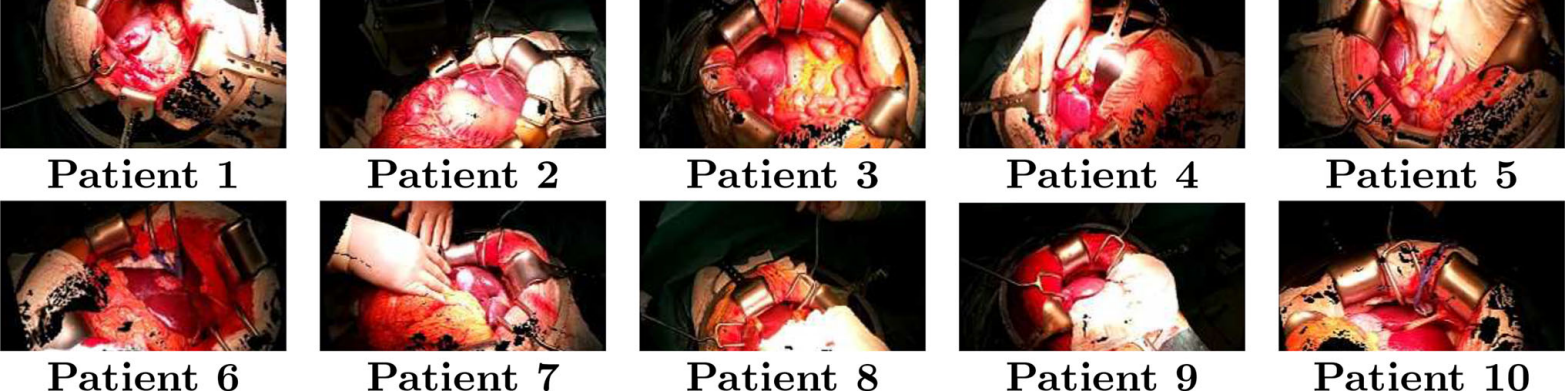
Sample depth-aligned RGB frames from each patient’s video. The frames illustrate the variations across patients

The HoloLens2 RGB camera delivers images at 1920 x 1080 pixels and up to 30 FPS. The Time-of-Flight (ToF) sensor, responsible for depth data, operates at a resolution of 320 x 288 pixels, 1–5 FPS (long-throw mode). Using hl2ss, we aligned RGB images with corresponding depth data by calibrating intrinsic and extrinsic sensor parameters, including the sensors’ positions and FOV (see Fig. 2).

The resulting dataset consisted of approximately 600 RGBD frames and camera poses per video. Camera poses described the position and orientation of the camera per frame. Annotations were performed by the first author, who supervised the data acquisition during surgeries and were cross-checked by a visceral surgeon with 36 years of experience.

#### Individual model comparisons

We evaluated the ESANet, SAM, DeAOT models individually on our clinical dataset to establish a baseline performance. Evaluation strategies were customized to align with each model’s operational framework.

**ESANet.** To evaluate prompt generation, a leave-one-patient-out approach was implemented, training on nine patients and testing on one. Moreover, training and validation sets were split using every 15th RGBD pair for validation. Early stopping was used when validation performance plateaued. Hyperparameters followed the original ESANet study [8].

**SAM.** To evaluate SAM independently of ESANet prompt quality, prompts were generated from ground truth annotations (see Sec. 2.1).

**DeAOT.** To evaluate DeAOT tracking of the videos, independent of SAM reference frame quality, we used ground truth annotation of the first frame of each video as reference frame. To obtain a statistical representation, we evaluated each video with totally five different start frames, each with an interval of 25 frames to the previous start frame.

#### Pipeline evaluation

Our multimodel pipeline was evaluated regarding segmentation accuracy (IoU) and temporal resolution (FPS). Temporal resolution included the average frame rate (FPS), accounting for inference times of all models and related processing steps. Both interval-based and scene-aware re-prompting strategies were tested to assess their impact on segmentation accuracy under dynamic clinical conditions. Interval-based re-prompting was tested using intervals from 50 to 10 frames in steps of 10 ($R_{50}$ – $R_{10}$), along with no re-prompting ($R_\infty$) and per-frame re-prompting ($R_1$). The scene-aware re-prompting was tested by monitoring the following parameters:

- **Segmented Area Size and Consistency**: Sudden changes in prediction size or consistency may cause signal tracking errors due to visibility changes or perspective changes. The size was tracked by measuring the percentage of segmented area per frame, and the consistency was assessed through IoU calculations between consecutive frames.
- **Depth Variation**: Variations in depth values within the segmented area may indicate tracking errors due to proximity changes or the inclusion of nearby structures. Depth stability was monitored by tracking the mean depth values ($\mu$ Depth) and their standard deviation ($\sigma$ Depth) within the segmented area in consecutive frames.
- **Camera Movement**: Camera movements, usually due to head motion while wearing the head-mounted AR device, can lead to errors in tracking and segmentation. Camera translation and rotation were tracked using camera pose data per frame.

The CUSUM algorithm was tested using five sensitivity levels by varying the threshold factor $\alpha$ in Eq. 4 from 2 to 10 in increments of 2. To reduce the influence of the initial frame, five starting frames, each 25 frames apart, were used per video. Statistical significance of the segmentation accuracy obtained by the best-performing scene-aware re-prompting strategy was tested against no re-prompting, using the Wilcoxon matched pairs signed-rank test. All evaluations were performed using an NVIDIA GeForce RTX 3090 GPU.

## Results

**Fig. 3:**
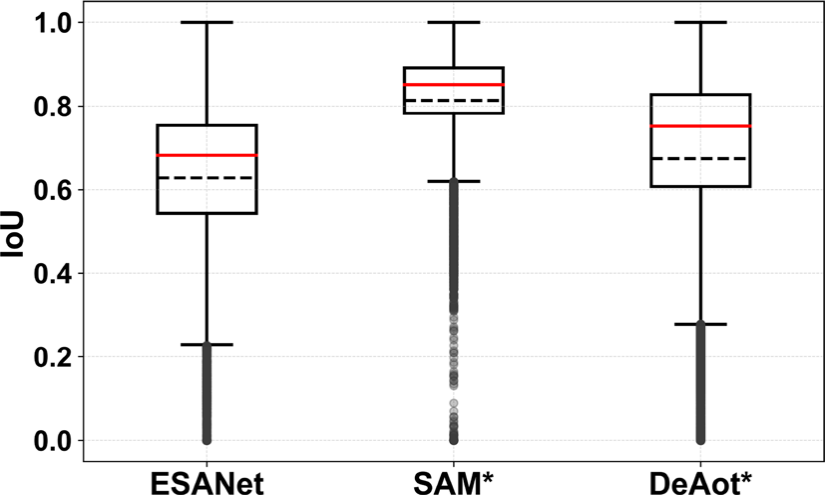
Comparison of segmentation accuracy (IoU) of the selected models for liver segmentation. Boxplots show IoU distributions, with medians (red line) and means (black dotted line). Models with optimal prompts/reference frames are marked by *

Figure 3 compares the segmentation accuracy (IoU) for the three individual models. For ideal prompt points, SAM achieved the highest median IoU of 85%, with low variability across all frames, indicating stable performance across patient videos and frames. DeAOT, using ground truth annotation for the first frame, reached a median IoU of 75%. ESANet, evaluated with a leave-one-patient-out strategy, showed a lower median IoU of 63%. The boxplots for ESANet and DeAOT reveal greater variability in IoU, suggesting that their performance is more influenced by specific frames and patient data.

Table 1 details interaction requirements and inference times for each model. SAM had the highest inference time, averaging 428.4 ms per frame, and requires user-generated prompts. DeAOT balanced accuracy and speed (84.1,ms/frame) but required a reference frame. ESANet was the fastest model (69.3,ms/frame) and required no user interaction, but had the lowest accuracy, with high variability.

**Table 1:** Comparison of the selected models considering accuracy, user interaction, and inference time

	ESANet	SAM*	DeAOT*
Median IoU	0.68	0.85	0.75
Average IoU	0.63±0.19	0.81±0.13	0.67±0.23
User Interaction	None	Prompts	Reference Frame
Inference Time / FPS	69.3 ms / 14.4 FPS	428.4 ms / 2.3 FPS	84.1 ms / 11.9 FPS

*Models were evaluated using ideal prompts and/or ideal reference frames according to ground truth annotations

**Fig. 4:**
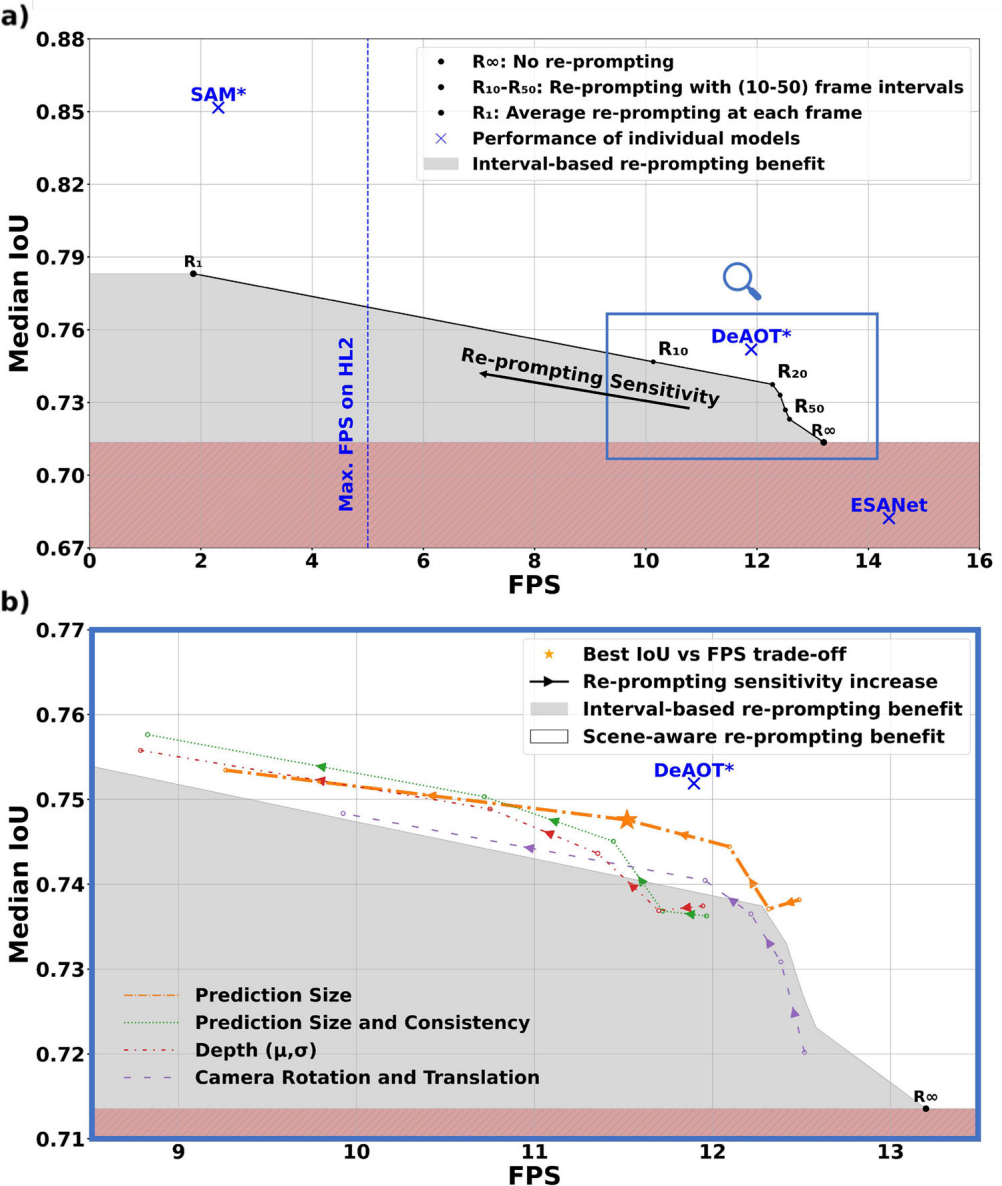
**a** IoU vs. FPS performance of our multimodel approach using interval-based re-prompting vs. individual models. Models with * used ideal prompts or reference frames. $R_{\infty }$: no re-prompting; $R_1$: re-prompting at each frame. **b** Zoomed-in view at the region of interest from (a), showing scene-aware re-prompting strategies as different lines. At $\alpha =4$, scene-aware prediction size re-prompting (marked by $\star$), nearly matched the DeAOT tracker performance with ideal frame initialization (IoU of 74.7% at 11.5 FPS). Lines represent various scene-aware re-prompting strategies. Gray area: performance lower than the interval-based strategy

Figure 4a compares individual model performances with our multimodel approach when using interval-based re-prompting. Results indicate that interval-based re-prompting generally enhances IoU but reduces temporal resolution. All results fall within a range framed by the baseline performances of SAM (highest IoU, lowest FPS), ESANet (lowest IoU, highest FPS), and DeAOT. Notably, most experiments achieved update rates that exceeded 2- 5 FPS and thus exceed the HoloLens2 depth sensor rate.

Without re-prompting ($R_{\infty }$ in Fig. 4), the multimodel approach achieves a median IoU of 71% at 13.2 FPS ($\sim$ 75 ms per frame). In contrast, re-prompting at every frame ($R_1$ in Fig. 4) raises median IoU to 78%, but reduces temporal resolution to 1.82 FPS due to SAM’s inference time.

Figure 4b presents results of the scene-aware re-prompting. Most scene-aware re-prompting techniques achieve a better trade-off between IoU and FPS than the interval-based method. To maintain clarity, only the best-performing strategies are shown in Fig. 4. The highest IoU (75.8%) was achieved by combining prediction size and consistency as re-prompting trigger at the highest re-prompting sensitivity ($\alpha$ = 2) with a temporal resolution of 8.3 FPS. However, the best trade-off between IoU and FPS was achieved using only prediction size as a re-prompting trigger. At various sensitivity levels, prediction size yields the best IoU vs. FPS trade-off at median IoU between 73.5% and 75.5% and temporal resolution between 9.2 and 12.5 FPS. At a re-prompting sensitivity $\alpha =4$, performance almost matched that of DeAOT, when initialized with an ideal reference frame (IoU of 74.7% at 11.5 FPS).

**Fig. 5:**
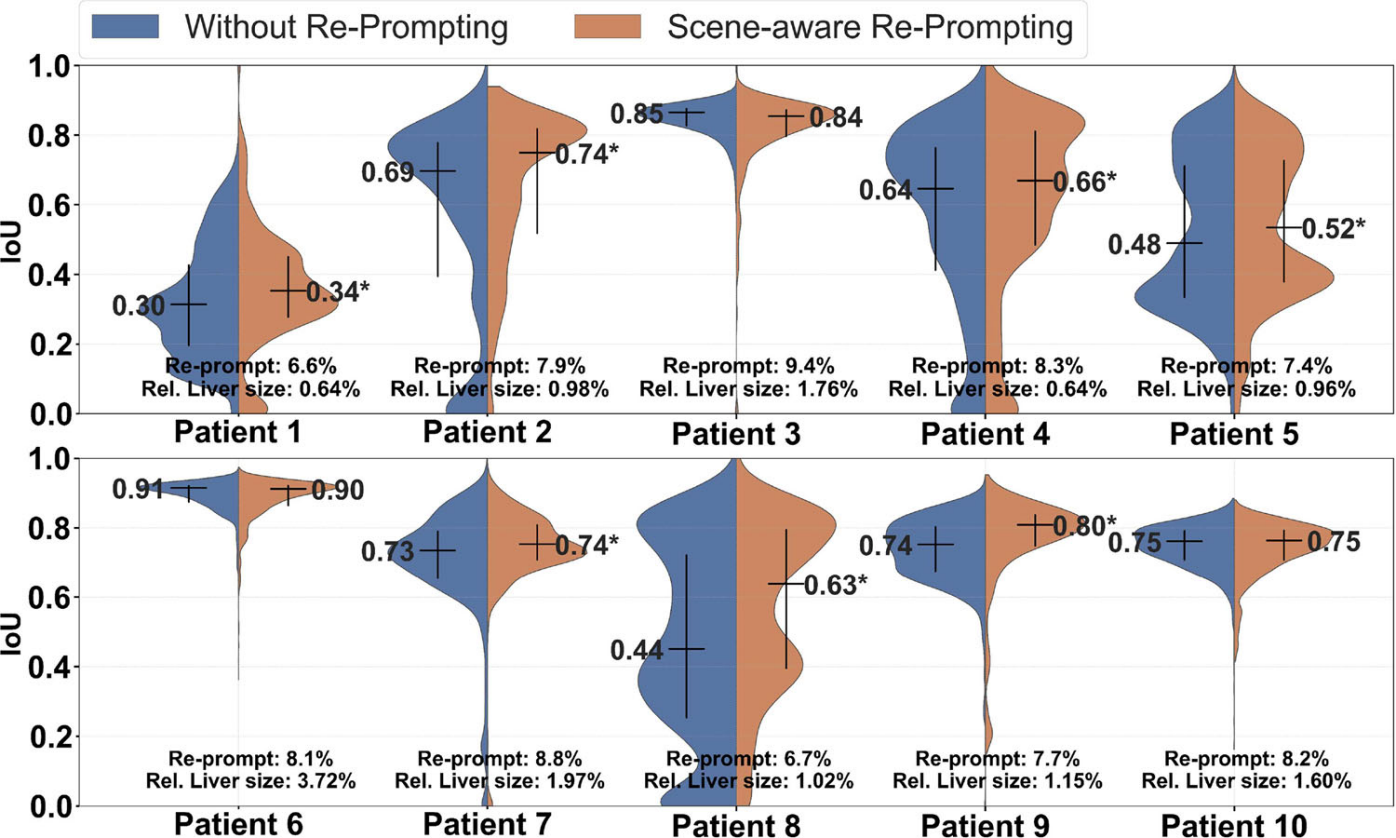
IoU comparison across patients for scene-aware re-prompting vs. no re-prompting. Results show that scene-aware re-prompting (triggered by prediction size at re-prompting sensitivity $\alpha =4$) maximizes segmentation accuracy. Scene-aware re-prompting leads to statistically significant improvements (marked by *) over no re-prompting ($p < 0.05$) for 7 of 10 patients. The average relative liver size was derived as the ratio of annotated liver pixels to the total image size

**Fig. 6:**
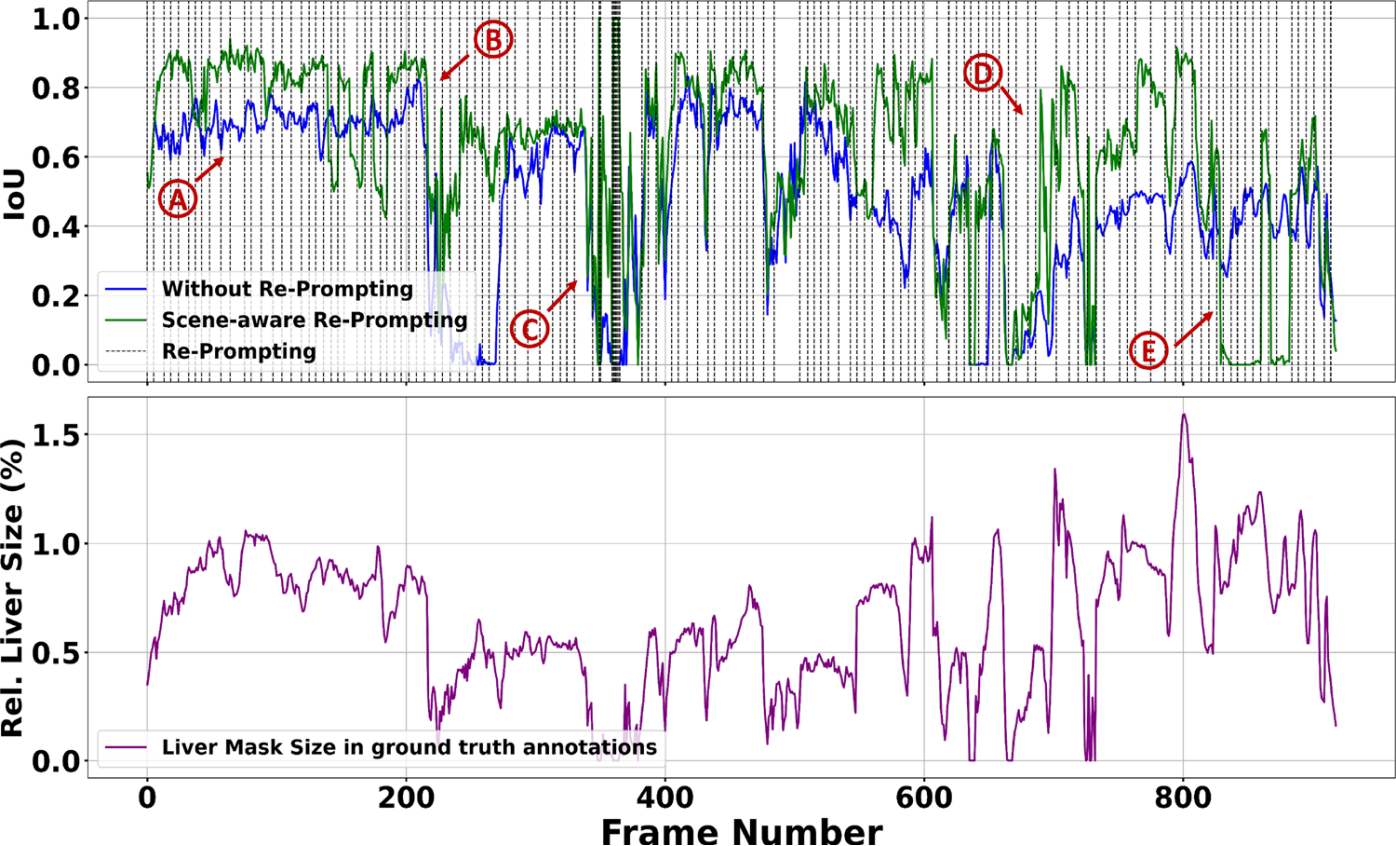
Effects of scene-aware re-prompting on segmentation accuracy (top) and variations in relative liver size for Patient 4 based on ground truth annotations (bottom)

Figure 5 compares IoU across patients at the optimal configuration (scene-aware re-prompting using parameter prediction size at re-prompting sensitivity $\alpha =4$) vs. no re-prompting. Moreover, Fig. 5 displays the share of frames that was used for re-prompting and the average relative liver size in each patient’s videos. The relative liver size was derived as the ratio of liver pixels to the total image size, according to ground truth annotations. Scene-aware re-prompting significantly improves segmentation performance compared to no re-prompting for 7 out of 10 patients ($p < 0.05$). Median IoU improvement was 4% on average, ranging from a maximum increase of 19% to a minimum of 1%. Patients with high initial performance without re-prompting (greater than 80%, e.g., Patients 3 and 6) did not show performance gains with scene-aware re-prompting. For Patients 3 and 6, the relative size of the liver surpasses that of other patients, as indicated by the mean relative liver size across all frames (see Fig. 5).

Figure 6 illustrates IoU scores with and without scene-aware re-prompting for all frames of Patient 4. Patient 4 was selected due to the large variety of scenes in the video, including periods of frequent liver visibility changes (periods B, C, and D) caused by surgeon interaction and camera movement, as well as a relatively static period (period A). The relative liver size, according to ground truth annotations, is shown to provide context for liver visibility across the frames. Excessive re-prompting can degrade performance, as observed in period E, where re-prompting during high occlusion or poor lighting caused segmentation inconsistencies.

## Discussion

This study reports initial results of AI-based liver segmentation and tracking using clinical data from open liver surgeries, captured with a head-mounted AR device. Our method leverages SAM for accurate segmentation while balancing accuracy and processing speed through scene-aware re-prompting.

The performance of fully supervised models like ESANet depends on the quantity and quality of training data, making data scarcity a persistent challenge. Although ESANet shows a low inference time (69.3 ms), it may be better suited for tasks where consistency is less critical, including prompting. Foundation models, including SAM, demonstrate high accuracy but incur high inference times (428 ms per frame), exceeding temporal requirements for AR-guided surgery. Semi-supervised models, including DeAOT balance speed and accuracy, still require user input, which limits their automation potential. Our fully automated multimodel approach is suitable for scenarios with deformations and occlusions that are common in abdominal surgery.

Our multimodel approach improves segmentation accuracy by 4% compared to the ESANet baseline, while sacrificing only 1 FPS even without re-prompting (Fig. 4). Re-prompting further improves segmentation accuracy. The CUSUM method controls the trade-off between segmentation quality and temporal resolution by adjusting the re-prompting sensitivity. In particular, re-prompting triggered by changes in prediction size produced results comparable to the ideally initialized DeAOT model (DeAOT*). The prediction size can be derived from the model output alone, demonstrating that our approach can succeed without additional sensor data, including the HoloLens2 IMU sensors.

Analysis of individual patient results revealed that lighting, occlusion, and liver-to-background contrast mostly influenced performance. Patients 3 and 6 demonstrated the highest performance due to clear visibility of the liver and the high contrast with adjacent structures (see Fig. 2 and Fig. 5). In contrast, for Patient 1 we observed the lowest performance due to liver occlusion and lighting. Additional training data for the ESANet model might improve the performance for Patient 1. Nonetheless, scene-aware re-prompting significantly ($p < 0.05$) enhanced segmentation accuracy over no re-prompting, independent of occlusion and lighting conditions. We attribute the increased segmentation accuracy to a mitigation of segmentation errors of the tracking model by scene-aware re-prompting, especially in dynamic video frames. The overall better performance of the SAM model contributes to the improvement in segmentation accuracy too. Given the absence of public datasets for open liver surgery, future research could investigate fine-tuning SAM using our dataset and transfer learning from laparoscopic data, e.g., Carstens et al. [20], to enhance segmentation performance for open liver surgery.

Scene-aware re-prompting substantially improved accuracy in periods with frequent liver visibility changes due to surgeon interaction and camera movement (periods B, C, D in Fig. 6). Scene-aware re-prompting furthermore improved accuracy during static periods (period A) by mitigating tracking errors. However, frequent re-prompting, as with the best trade-off configuration (scene-aware re-prompting triggered by prediction size with $\alpha =4$), can degrade performance, especially when triggered during unsuitable frames (e.g., high occlusion or poor lighting, as shown in period E in Fig. 6). Balancing re-prompting sensitivity is crucial to achieve improved performance without interrupting the tracking process. The CUSUM algorithm relies on features from previous frames. As a result, the algorithm is vulnerable to early frame segmentation errors that can propagate to later frames. Future work could dynamically adjust re-prompting sensitivity (via $\alpha$) based on observed frames or incorporate uncertainty-aware segmentation methods to reduce error propagation [21].

The optimal FPS for AR-guided surgery depends on multiple factors, including sensor capabilities, real-time computational processing, and clinical workflow constraints. Dynamic surface tracking and non-rigid registration are crucial for AR guidance in soft tissue surgery. In clinical settings, processing time for both, tracking and registration, depends on hardware limitations, including the RGBD sensor frame rate, computational demands, and potential data transmission latencies. The HoloLens is widely used in AR-guided surgery applications, where computational offloading, as applied in the present work, is reasonable within an operating room [6]. In our setup, the ToF long-throw mode of Hololens2 was operated at 5 FPS for data acquisition. Hololens2 furthermore offers an Articulated Hand Tracking (AHAT) mode, which provides higher frame rates (up to 45 FPS), but is limited to short-range operation (up to 1 m). Both long-throw and AHAT modes are subject to depth-sensing inaccuracies inherent to ToF technology [22]. Future implementations could explore the AHAT mode and other sensor technologies to further enhance temporal resolution. Even at the currently limited frame rates AR systems can already create clinical benefits [23]. Our approach offers a flexible balance between segmentation accuracy and processing speed and could be implemented on different hardware configurations. In particular, our approach optimizes surface tracking accuracy and frame rate to meet the computational requirements associated with non-rigid registration.

Future clinical evaluations could investigate performance and real-time applicability of the markerless AR guidance pipeline as well as its impact on surgical efficiency and intraoperative decision-making.
